# Agronomic strategies to enhance the early vigor and yield of maize part II: the role of seed applied biostimulant, hybrid, and starter fertilization on crop performance

**DOI:** 10.3389/fpls.2023.1240313

**Published:** 2023-11-03

**Authors:** Luca Capo, Alessandro Sopegno, Amedeo Reyneri, Gergely Ujvári, Monica Agnolucci, Massimo Blandino

**Affiliations:** ^1^ Department of Agriculture, Forest and Food Sciences, University of Turin, Grugliasco, Italy; ^2^ Department of Agriculture, Food and Environment, University of Pisa, Pisa, Italy

**Keywords:** corn, early vigor, grain yield, seed treatment, diammonium phosphate, *Bacillus amyloliquefaciens*

## Abstract

Maize cropping systems need to be re-designed, within a sustainable intensification context, by focusing on the application of high-use efficiency crop practices, such as those that are able to enhance an early plant vigor in the first critical growth stages; such practices could lead to significant agronomic and yield benefits. The aim of this study has been to evaluate the effects of the cultivation of hybrids with superior early vigor, of the distribution of starter fertilizers at sowing, and of the seed application of biostimulants on promoting plant growth and grain yield in full factorial experiments carried out in both a growth chamber and in open fields. The greatest benefits, in terms of plant growth enhancement (plant height, biomass, leaf area) and cold stress mitigation, were detected for the starter fertilization, followed by the use of an early vigor hybrid and a biostimulant seed treatment. The starter fertilization and the early vigor hybrid led to earlier flowering dates, that is, of 2.1 and 2.8 days, respectively, and significantly reduced grain moisture at harvest. Moreover, the early vigor hybrid, the starter NP fertilization, and the biostimulant treatment increased grain yield by 8.5%, 6.0%, and 5.1%, respectively, compared to the standard hybrid and the untreated controls. The combination of all the considered factors resulted in the maximum benefits, compared to the control cropping system, with an increase in the plant growth of 124%, a reduction of the sowing-flowering period of 5 days, and a gain in grain yield of 14%. When choosing the most suitable crop practice, the diversity of each cropping system should be considered, according to the pedo-climatic conditions, the agronomic background, the yield potential, and the supply chain requirements.

## 1 Introduction

Maize (*Zea mays* L.) is the first commodity, in terms of the worldwide annual production of dry grain, and it plays a key role in global agri-food systems, since it is used for food and feed consumption and several non-food purposes, such as the production of starch, chemical compounds, and energy. The global maize production has surged in recent decades, driven by the increasing demand, and by a combination of yield increases and area expansions (Erenstein et al., 2022). Furthermore, the global use of maize is destinated to increase, although the prospects for further area expansions are limited, thus highlighting the need to innovate cropping systems to further increase their production capacities and enhance a sustainable intensification of maize-based systems.

One of the agronomic strategies that has allowed maize yields to be increased in temperate growing areas, but also the qualitative and sanitary traits, is the progressive ability to anticipate the sowing period in spring, which leads to an increase in the length of the growing cycle and avoids possible heat and drought stress during the flowering or the ripening (Van Roekel and Coulter, 2011; Waqas et al., 2021). Moreover, in order to prevent the negative impact of cold and rainy periods during the first vegetative stages, which can limit the advantages of early sowing, this practice is often associated with the use of good early vigor hybrids and also practices applied during seed crop growing which support high vigor in the next generation, such as the application in bands close to the seed furrows of starter fertilizers, mainly those containing nitrogen (N) and phosphorus (P) (Kaiser et al., 2016; Reis et al., 2022). Since a fast and uniform plant growth plays a key role in achieving the full potential yield of maize and in promoting higher resource use efficiencies, these crop practices are adopted to efficiently limit a delay in maize development, which could reduce the benefits of early sowing, thereby significantly contributing to the profitability of the crop.

Biostimulants represent another promising developing strategy to enhance plant development and to reduce biotic and abiotic stresses, particularly during the most critical growth stages of the crop (du Jardin, 2015; Cardarelli et al., 2022). Biostimulants, which have been defined by the European Biostimulant Industry Council as “substances and/or micro-organisms whose function is to stimulate natural processes that enhance nutrient uptake, nutrient use efficiency, tolerance to abiotic stress, and crop quality” can offer an eco-friendly option to reduce the use of external inputs, such as mineral fertilizers, and increase the sustainability of cropping system, without a reduction of crop productivity (Gupta et al., 2021; Li et al., 2022). The application of specific species of plant growth promoting bacteria (PGPB) and/or substances that are readily available and rapidly assimilated by the rhizosphere microbial community has been proposed, as biostimulants and biofertilizers, as they could lead to beneficial effects on the crop, as a consequence of the production of plant growth regulators (phytohormones), antifungal secondary metabolites, and an increase in plant nutrient uptake (Tahir et al., 2018; Aloo et al., 2022). In addition to a direct effect on plant growth, such crop practices as the choice of hybrids with different early vigor, or the application of starter fertilizers or biostimulants at sowing, could also influence the rhizosphere bacterial richness and functionality (Favela et al., 2021). Since plants rely on their microbiota for several functions, including nutrient acquisition and protection against stress (Compant et al., 2019), the effect of enhancing rhizosphere microbiota through the application of crop practices could lead to further indirect benefits for the early development of plants, particularly in the most critical early vegetative stages. In part I of the present study (Ujvári et al., 2023), the role of maize genotype, NP starter fertilization, a biostimulant seed treatment, and their interaction, was studied on native rhizosphere microbiota during the early growth phases of maize in a growth chamber experiment. The diversity and composition of the rhizosphere bacterial communities was mainly affected by the genotype, when hybrids with different early vigor were considered, and by the starter fertilization. Moreover, the collected data highlighted that the use of an early-vigor hybrid, and NP fertilization, also increased the abundance of specific bacterial taxa (*Stenotrophomonas*, *Lysobacter*, *Massilia*, *Paenibacillus* and *Flavobacterium*), which have been reported to be able to provide a significant plant growth promotion (Hayward et al., 2010; Kour et al., 2019; Li et al., 2021). Moreover, the collected data also showed that a biostimulant seed application resulted in a significant enhancement of the rhizosphere bacterial community, but, interestingly, the microbiota of the standard hybrid was affected more than the early vigor one by the biostimulant treatment, thus showing an interaction among the compared crop practices.

To the best of our knowledge, the effects of a biostimulant application on crop development, and the related benefits, in terms of both grain yield and quality, have not been adequately investigated for crop plants, by considering a comparison and interaction with other agronomic practices that are able to enhance plant vigor. Moreover, the benefits associated with the application of PGPB have generally been reported for marginal environments (Li et al., 2022), while their contribution to intensive systems has not yet been clarified.

Considering the current need to re-design maize cropping systems in order to enhance their inputs and use efficiency, and to lead to a more sustainable production, the aim of the present study, part II of the work, has been to evaluate and compare the agronomic and yield effects of different crop practices that are able to enhance the early plant vigor. The effect of genotype early vigor, NP starter fertilizer, and a biostimulant seed treatment on maize development in the early stages, and the consequent effect on the crop cycle length, and on the grain yield and quality, has been studied in depth in both a growth chamber experiment and open field experiments, considering factorial combinations of the compared agronomic factors in different locations (i.e. in different soils) and in different growing seasons.

## 2 Materials and methods

A growth chamber experiment was set up to investigate the effects of i) various maize hybrids, considering genotypes with different early vigor, ii) NP starter fertilization in seed furrows and iii) a biostimulant, based on a PGPB and plant extract, applied to seeds, in promoting maize plant development in the early stage under cold conditions. The same experimental design was replicated under open field conditions in the 2018 and 2019 growing seasons, to demonstrate the role of different agronomic factors in favoring the early growth of maize in a real environment, and to quantify the benefits in terms of grain yield and quality.

### 2.1 Growth chamber experiment

#### 2.1.1 Experimental design

A detailed description of the management of the growth chamber experiment is reported in part I of the present work (Ujvári et al., 2023). Briefly, an amount of sixteen kilograms of natural silt loam sub-alkaline soil (Typic Ustifluvents, USDA classification), was weighed and placed, after mixing it thoroughly, into each plastic pot (27 cm long x 24 cm wide x 28 cm high). The soil was collected from the surface layer (0.2 m) of a field in the University of Turin experimental station, located in Carmagnola, North-West Italy (44° 53’ N, 7° 41’ E; elevation 245 m). The soil was characterized by a medium cation-exchange capacity (C.E.C.), low organic matter, K, and P contents, and medium nitrogen availability. More information on the physical and chemical parameters of the soil are reported in **Table 1**. The soil was not air dried, sieved, sterilized or mixed with quartz sand or any other materials.

**Table 1:** The main physical and chemical characteristics of the topsoil (0-30cm) in the Carmagnola and Poirino experimental sites.

Parameters	Measurement units	Carmagnola ^1^	Poirino
Geographical coordinates		44°53’ N, 7°41’ E	44°56’ N, 7°52’ E
Soil type		Typic Ustifluvent	Fluventic Haplustepts
Soil texture		Silt loam	Silt loam
Sand (0.05 - 2 mm)	g kg^-1^	272	233
Silt (0.002 - 0.05 mm)	g kg^-1^	680	696
Clay (< 0.002 mm)	g kg^-1^	48	70
pH (H_2_0)		7.9	6.5
Total carbonate (CaCO_3_)	g kg^-1^	12	4
Organic matter	g kg^-1^	18.2	19.8
C/N		8.6	9.2
Cation Exchange Capacity (C.E.C.)	cmol(+) kg^-1^	11.0	13.1
Total Nitrogen	g kg^-1^	1.23	1.26
Exchangeable Potassium	mg kg^-1^	52	70
Olsen Phosphorus	mg kg^-1^	6	73

^1^ the Carmagnola soil was used in the growth chamber experiment.

The compared treatments were factorial combinations of:

- maize hybrids; considering genotypes with different early vigor after emergence, but with a similar growing cycle (FAO maturity class 600, 130 maturity days),
   ○ a standard hybrid, with conventional early vigor (LG30600, Limagrain Europe, Saint-Beauzire, France),
   ○ an early-vigor hybrid, with a rapid growth in the first vegetative stages (LG31630, characterized by the Rapid’START trait, Limagrain Europe);
- NP starter fertilization,
   ○ unfertilized control (unfertilized),
   ○ sub-surface starter fertilization (NP), whereby 27 kg N ha^-1^ and 69 kg P_2_O_5_ ha^-1^ were applied as diammonium phosphate (DAP, 18-46%, for N and P_2_O_5_, respectively w/w) and placed in bands close to the maize seed furrow;
- biostimulant seed treatment,
   ○ untreated control (no biostimulant),
   ○ biostimulant seed application (biostimulant, commercial product Starcover, Limagrain Europe), based on a mixture of a bacterium, *Bacillus amyloliquefaciens* strain IT-45 (Rise P^®^ Lallemand Plant Care, Castelmaurou, France) and a leguminous plant extract *Cyamopsis psoraloides* (AgRHO^®^ GSB30 Solvay, Clamecy, France), which works as coating film to favor germination by channeling water from soil to seed.

The adopted experimental pot design was a completely randomized block design with three replications for each treatment. All the maize seeds were treated with a fungicide mixture of prothioconazole (100 g L^-1^) and metalaxyl (20 g L^-1^), which was applied, at 15 mL, to 50,000 seeds (Redigo® M, Bayer Crop Science S.r.l., Monheim am Rhein, Germany). The shape, dimensions and weight of the maize seeds were chosen carefully to reduce the variability of the seedling vigor. Four maize seeds were sown by hand and equally distributed in each pot at a depth of 2 cm. After germination, only 2 plants per pot were maintained, while the other 2 plants were manually removed at the first leaf stage to ensure the conventional field density. The starter NP fertilizer was manually placed in band at a distance of 5 cm from the maize seed furrows, and at a depth of 10 cm. No other fertilizers were applied before or after sowing. The pots were placed in a controlled growth chamber, with 50% relative humidity, a 12 h photoperiod, 700 μmol m^-2^ s^-1^ photosynthetically active radiation (PAR), and a 14/17°C (night/day) air temperature range (**Table S1**).

#### 2.1.2 Crop measurements

The plant height was measured in centimeters, from the ground level up to the collar of the tallest fully developed leaf, at the 2 (growth stage, GS, 12, 21 days after sowing, DAS), 3 (GS13, 35 DAS), and 5 (GS15, 47 DAS) leaf stages (BBCH scale, Lancashire et al., 1991). At the same time, the Leaf Area (LA) was estimated according to Ruget et al. (1996). The chlorophyll leaf content was measured at the 4-leaf stage (GS14), using a SPAD 502Plus-chlorophyll meter® (Konica-Minolta, Osaka, Japan), and expressed as SPAD units. Two SPAD readings were taken around the midpoint of each unrolled leaf for both maize plants in each pot and averaged. The spectral signature (from 350 to 2500 nm) of each unfolded leaf was acquired at GS14 using a NaturaSpec™ RS-5400® Portable spectroradiometer (Spectral Evolution, Haverhill, USA). We used a leaf clip bundle (Spectral Evolution, Haverhill, USA), which was specifically designed for leaf reflectance measurements, and which has an integrated light source and white reference, to collect sample spectra. One observation was considered for each maize leaf for both pot plants. The collected data allowed the vegetative indices related to the P (normalized phosphorus content index, NPCI, according to Mahajan et al. (2014) and carotenoid (Carotenoid Reflectance Index, CRI_700_, according to Gitelson et al. (2001) leaf content to be collected, according to the following formulas:


$$\text{NPCI}=\;\frac{\left({\text{R}}_{1080}-{\text{R}}_{1460}\right)}{\left({\text{R}}_{1080}+\;{\text{R}}_{1460}\right)}$$

and


$${\text{CRI}}_{700}\;={\left({\text{R}}_{510}\right)}^{-1}-\;{\left({\text{R}}_{700}\right)}^{-1}$$

where R is the reflectance at the corresponding subscripted wavelength (nm).

The normalized difference vegetation index (NDVI) of each pot was measured, at the 5-leaf emission stage (GS15, 47 DAS), by means of a pistol grip mounted onto the RS-5400 spectroradiometer and elaborated with DARWin SP Data Acquisition® software (Spectral Evolution, Haverhill, USA). The instrument was held at a height of 1.36 m above each single pot, using a 25-degree exercise fiber cone, to detect the spectral signature (in the 350-2500 nm range) of both plants sown in each pot. This assessment was carried out under standard conditions, placing the pot in a room with only artificial light (ILM-550 Tungsten-Halogen Light Sources) and using an opaque plastic black cloth placed on the floor and on the surface of the pot to only detect the plant reflectance. An NDVI measurement permits the development of the crop canopy to be quantified, since the obtained values are proportional to the maize biomass (Capo et al., 2020). The shoots and root system of each plant were collected at 49 DAS, after cutting the maize shoots at the collar and gently removing the soil by hand. The shoot and root dry biomasses were determined after their biomass had been oven dried at 105°C for 24h. The obtained data were expressed in grams per plant of dry weight (d.w.).

### 2.2 Field experiments

#### 2.2.1 Site and treatments

Two field experiments were set up in the 2018 and 2019 growing seasons in two locations in North-West Italy: Carmagnola, in the same soil used in the previous growth chamber experiment, and Poirino. The main physical and chemical characteristics of these soils are reported in **Table 1**. The greatest differences between the soil in the considered locations concerned the pH and the available P content (Olsen et al., 1954): Carmagnola is characterized by a sub-alkaline soil with a low P content, while the Poirino soil is sub-acidic and has a high P availability. The 2019 experiment was performed in a new area, adjacent to the one used in 2018, in both sites to avoid carry-over effects resulting from the treatments. The daily air temperatures and precipitations were measured at Regione Piemonte meteorological stations located near (within 5 km) each site. The soil temperature was measured, during the crop emergence and seedling stages, using a GP-4020 Tinytag Plus 2 devices, with a 10 cm thermistor probe (Gemini Data Loggers Ltd, Chichester, UK), which was placed along the seed rows. The same experimental design as the one adopted in the growth chamber trial was adopted at each location and in each year, that is, a factorial combination of 2 hybrids (standard vs early vigor), 2 fertilizations close to the sowing furrows (unfertilized vs NP), and 2 seed treatments (no biostimulant vs biostimulant). The treatments were assigned to experimental units in each site using a completely randomized block design, with four replicates. Each plot consisted of 4 rows 0.75 m apart; the plot length and the alleys between the plots were 12 and 1 m, respectively. All the measurements were conducted in the two middle rows. The starter fertilizers were placed at a distance of 5 cm from the side of the seed furrows, using a calibrated granular dispenser attached to the planter (Monosem NG, Largeasse, France), at a depth of 10 cm from the soil surface. The conventional crop technique of the growing areas was adopted. Briefly, in Carmagnola, sowing was carried out after autumn ploughing (at a 0.3 m depth), followed by disk harrowing, while, in Poirino, a seedbed was prepared by means of a sub-soiler, followed by power harrowing. The previous crop was maize in each field. The sowing density was 8.0 plants per m^2^. The sowing and harvest dates are reported for each year and site in **Table 2**. A granular soil insecticide (tefluthrin 0.183 kg AI ha^-1^) was applied at sowing to the seed furrows to protect the seedlings from injury by ground insects (Force® Ultra 1.5%, Syngenta Crop Protection S.p.A.). The weed control was conducted, at pre-emergence, with mesotrione (0.15 kg AI ha^-1^), S-metolachlor (1.25 kg AI ha^-1^), and terbuthylazine (0.75 kg AI ha^-1^) (Lumax®, Syngenta Crop Protection S.p.A., Basel, Switzerland), and at post-emergence with nicosulfuron (36.8 kg AI ha^-1^), rimsulfuron (9.2 kg AI ha^-1^), and dicamba (220 kg AI ha^-1^) (Principal® Mais, Corteva Agriscience, Cremona, Italy). Before sowing, 157 kg ha^-1^ of K_2_O was applied (as potassium chloride, 60% K_2_O w/w), but no other N or P fertilizations were distributed, except for the starter in bands close to the seed furrows. A total of 230 kg ha^-1^ of N was applied at approximately the 8^th^ unfolded leaf growth stage (GS18), using urea (46% N w/w), for all the treatments at each site and in each year. Irrigation was carried out, at both sites, by means of the sprinkler method, according to the conventional farm management system in force in the experimental area, to avoid any drought stress until physiological maturity (GS87).

**Table 2:** The main agronomic information, cumulative monthly rainfall, air growing degree days on a 10°C basis (GDDs), medium temperature of the soil, and GDDs during the maize crop cycle in the 2018-2019 period at Carmagnola and Poirino (North-West Italy).

Agronomic information		2018		2019	
		Carmagnola	Poirino	Carmagnola	Poirino
Sowing date		April 20	April 26	March 22	March 21
Harvesting date		September 25	October 1	September 16	September 24
Rainfall (mm)	March	103	105	25	6
	April	116	82	184	85
	May	310	183	272	103
	June	14	52	54	25
	July	74	85	177	120
	August	6	21	119	86
	September	35	51	82	63
	Sowing - 6 leaves	244	184	402	139
	Sowing - Harvest	439	397	884	482
Air GDDs^1^ (C°-day)	March	5	9	58	48
	April	151	155	129	80
	May	255	238	195	165
	June	372	360	417	395
	July	462	463	496	466
	August	457	467	457	436
	September	327	345	318	286
	Sowing - 6 leaves	290	275	265	211
	Sowing - Harvest	1942	1912	1901	1798
Medium temperature of the soil (C°)	Sowing - 6 leaves	20.1	18.8	14.8	15.2
Soil GDDs (C°-day)	Sowing - 6 leaves	353	307	288	327

#### 2.2.2 Early vigor

Different assessments were performed to establish plant vigor in the early growth stages. The NDVI was measured from the 3-leaf stage (GS13) until tassel emission (GS55) for each plot using a hand-held optical sensing device, GreenSeekerTM® (Trimble©, Sunnyvale, California, USA). The instrument was held approximately 60 cm above each single maize row, and its effective spatial resolution was 0.75 m × the full length of the plot (12 m). This assessment was performed approximately every 7 days, on the two middle rows of each plot. The NDVI measurement helped to quantify the development of the crop canopy throughout the season, since low values refer to naked soil, while high values are proportional to the maize biomass. The Area Under Canopy Development Curve (AUCDC) (Capo et al., 2020) was calculated during the vegetative stage for each treatment, starting from the NDVI measurement for each observation date and using the following formula:


$$\text{AUCDC}=\;\sum_{i}^{\text{n}-1}\left\{\left[\left(R_{i}+{\text{R}}_{\text{i}+1}\right)/2\right]\;\left({\text{t}}_{\text{i}+1}-\;t_{i}\right)\right\}$$

where R is the NDVI value, t is the time of observation, and n is the number of observations.

Plant height was recorded at approximately the 4-leaf stage (GS14) and at stem elongation (GS33, approximately 3 detectable nodes) by measuring 20 consecutive randomly selected plants within the central two rows of each plot. Plant height was measured, in centimeters, from the ground level up to the collar of the tallest fully developed leaf (GS14), or from the ground level up to the tallest detectable node (GS33). The number of days from sowing until the day when > 50% of the plants in the two central rows of each plot had reached the beginning of ear flowering (GS62) was recorded. This parameter was expressed as DAS.

#### 2.2.3 Grain yield and yield parameters

Ears were collected by hand at harvest maturity from 4.5 m^2^ in the two central rows of each plot to quantify the grain yield and to obtain a representative sample. The harvesting was performed when the grain moisture content was between 23 and 30%, according to the conventional harvesting practice in the growing areas. The collected ears from each plot were counted to record the density per square meter of the fully developed ears. The number of kernel rows and the number of kernels per row were also counted on 7 of these randomly selected and de-husked ears, and the theoretical amount of kernels per square meter (KSM) was then calculated by multiplying the average number of kernels per ear by the number of ears m^-2^ (Blandino et al., 2022). All the collected ears were shelled using an electric sheller. The kernels from each plot were mixed thoroughly to obtain a random distribution. Grain moisture was analyzed using a GAC2100 Dickey-John grain analyzer (Auburn, IL, USA). The grain yield results were adjusted to a commercial moisture level of 14%. Aliquots of 5 kg were taken and dried at 60°C for 72 hours to reduce the kernel moisture content to 10%. Two hundred dry kernels were randomly collected, considering only whole seeds, and weighed using an electronic balance to assess the thousand kernel weight (TKW).

### 2.3 Statistics

The Kolmogorov–Smirnov normality test and the Levene test were carried out to verify the normal distribution and homogeneity of variances. An analysis of variance (ANOVA) was performed for the growth chamber data (plant height, LA, vegetative indices, shoot and root biomass) and field experiment data (plant height, AUCDC vegetative index, date of flowering, grain moisture, grain yield, and yield parameters), with the maize hybrid, starter fertilization, and biostimulant seed treatment being considered as independent factors. ANOVA was carried out separately in the field experiment for each compared soil and for each year. Multiple comparison tests were performed, according to the Ryan-Einot-Gabriel-Welsh F (REGW-F) test, on the treatment means. Statistical data analysis was carried out with the SPSS software package, version 27.0.

## 3 Results

### 3.1 Growth chamber experiment

**Figure 1:**
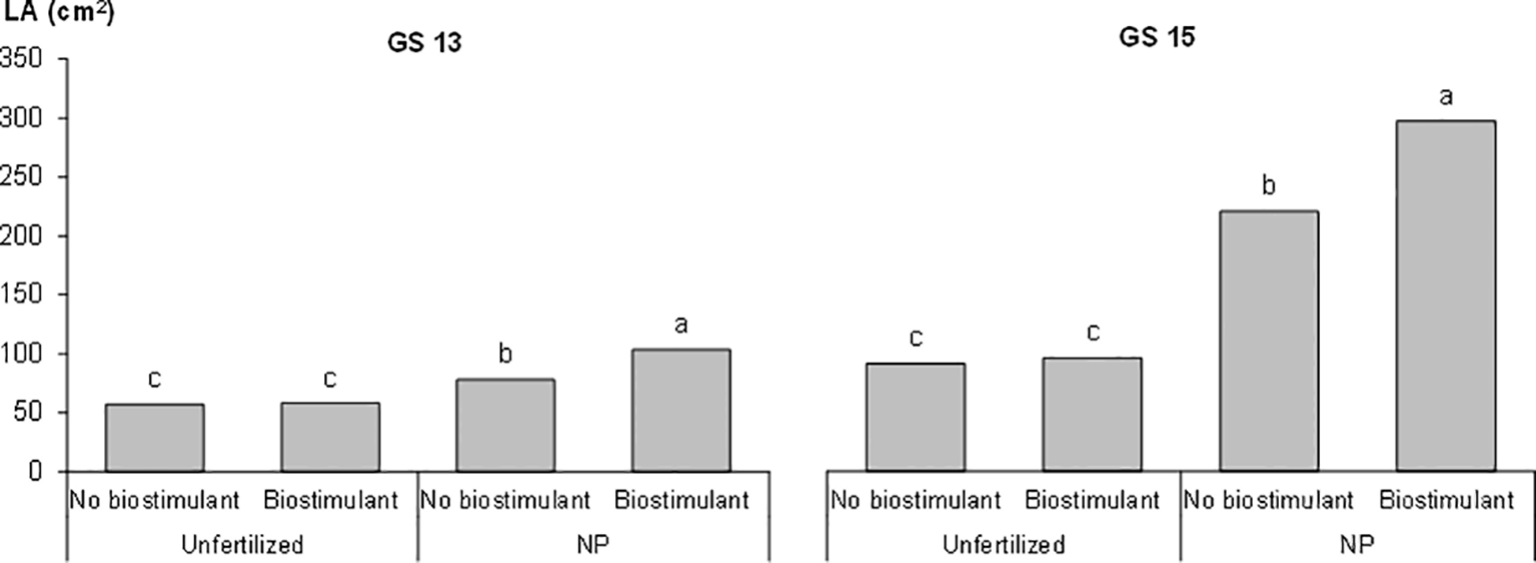
Effects of the starter fertilization and the biostimulant seed treatment on the maize leaf area (LA) at the three- (GS13) and five-leaf (GS15) emission stages in the growth chamber experiment. Bars with different letters are significantly different (*p*-value<0.05), according to the REGW-F test.

**Figure 2:**
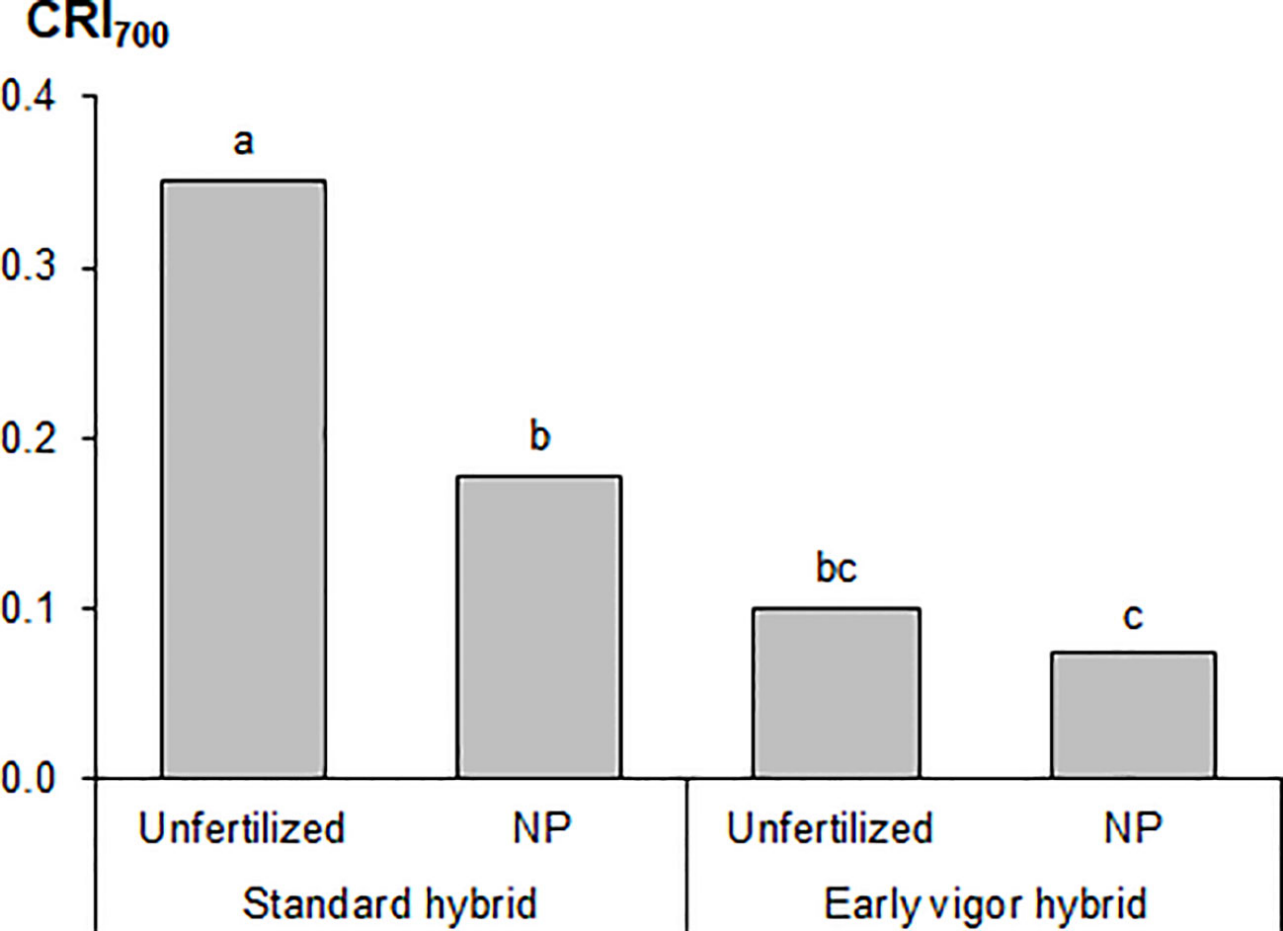
Effects of the maize hybrid and the starter fertilization on the carotenoid reflectance index (CRI_700_) at the four-leaf emission stage (GS14) in the growth chamber experiment. Bars with different letters are significantly different (*p*-value<0.05), according to the REGW-F test.

The plant height and LA of the plants, as measured during the first vegetative stages, that is, from GS12 to GS15, are reported in **Table 3**. The early vigor hybrid showed a significantly higher plant height than the standard one at GS15 (+10%); although a significantly higher LA was already observed at the 2-leaf stage for this genotype and it was maintained in the following growth stages. The starter NP fertilization led to the first significant increase in plant height at GS13 (+30%, compared to unfertilized control), which resulted in an increased advantage at GS15 (+53%), while a significant positive effect of starter fertilization, in terms of LA, was already detected at GS12. The biostimulant seed treatment led to a significantly higher plant height and LA than the untreated control (no biostimulant), from GS13. As far as the NDVI value at GS15 is concerned, the greatest benefits, in terms of plant growth and development, were on average observed for the starter NP fertilization (+60%), followed by the biostimulant seed treatment (+11%), and the use of an early vigor hybrid (+8%). The interaction between starter fertilization and seed treatment was significant: the biostimulant seed treatment resulted in a significant increase in LA, albeit only when the NP starter fertilizer was applied at both GS13 and GS15 (**Figure 1**). All the compared agronomic factors resulted in a significant effect on the vegetative indices related to leaf greenness or to the manifestation of plant stress, which was associated with red-yellow colors at GS14 (**Table 4**). The maize hybrid and fertilization significantly affected CRI_700_ (red-yellow color). The starter NP fertilization resulted in the strongest effect, as it increased the chlorophyll content (green color) by 42% and reduced CRI_700_ (red-yellow color) by 44%. The early vigor hybrid showed a lower CRI_700_ (-67%) and chlorophyll content (+5%) values than the standard one. A significant interaction was reported between hybrid and fertilization on CRI_700_: the NP starter fertilization significantly decreased this vegetative index in the standard hybrid control, while no significant differences were detected for the early vigor one (**Figure 2**). Conversely, the biostimulant seed treatment did not result in a significant effect on the chlorophyll content or on CRI_700_. All the compared factors influenced NPCI to a great extent: on average, the starter NP fertilization resulted in the highest increase (+77%) of this index, and this was followed by the hybrid (+23%), and by the biostimulant seed treatment (+22%). The NP starter fertiliser and biostimulant seed treatment significantly affected both the shoot and root biomass measured at GS15 (**Table 4**). The application of the NP starter fertilizer at sowing showed the greatest effect: on average, the dry biomass increased by 4.6 times and 2.2 times for the shoots and roots, respectively. The biostimulant seed treatment significantly enhanced maize biomass production, that is, by 21% (shoots) and by 20% (roots), compared to the untreated control (no biostimulant). The interaction between the starter fertilization and the seed treatment was significant for the shoot biomass: the application of a seed biostimulant significantly improved the shoot biomass (+24%), albeit only under the NP fertilized conditions (data not shown).

**Table 3:** Effects of the hybrid, starter fertilization, and biostimulant seed treatment on the early vigor of maize, expressed as plant height and leaf area (LA) at the two- (GS12), three- (GS13) and five-leaf (GS15) emission stages, and the Normalized Difference Vegetation Index (NDVI) value, in the growth chamber experiment.

Factor	Source of variation	Plant height (cm)			LA (cm^2^)			NDVI
		GS12	GS13	GS15	GS12	GS13	GS15	GS15
Hybrid (H)	Standard	5.1 a	7.9	11.8 b	26.3 b	68.9 b	164.8	0.250 b
	Early vigor	4.4 b	8.3	13.0 a	29.2 a	76.4 a	181.1	0.271 a
	*p*-value	0.024	0.530	0.006	0.006	0.029	0.053	0.013
Fertilization (F)	Unfertilized	4.5	7.1 b	9.8 b	27.0 b	57.5 b	93.3 b	0.200 b
	NP	5.0	9.2 a	15.0 a	28.7 a	89.6 a	254.1 a	0.320 a
	*p*-value	0.078	<0.001	<0.001	0.031	<0.001	<0.001	<0.001
Seed treatment (S)	No biostimulant	4.7	7.9 b	12.1 b	27.5	68.0 b	160.9 b	0.247 b
	Biostimulant	4.8	8.4 a	12.8 a	28.2	78.2 a	186.5 a	0.274 a
	*p*-value	0.586	0.004	0.002	0.174	<0.001	<0.001	0.006
H x F	*p*-value	0.951	0.073	0.085	0.815	0.612	0.524	0.053
H x S	*p*-value	0.166	0.218	0.574	0.869	0.673	0.406	0.910
F x S	*p*-value	0.416	0.009	0.106	0.112	<0.001	<0.001	0.460
H x F x S	*p*-value	1.000	0.405	0.661	0.398	0.688	0.672	0.392

Means followed by different letters are significantly different. The level of significance (*p*-value) is shown in the Table.

**Table 4:** Effects of the hybrid, starter fertilization, and biostimulant seed treatment on the carotenoid reflectance index (CRI_700_), on the chlorophyll content measured by means of the SPAD device, and on the Normalized Phosphorous Content Index (NPCI) of the leaves at the 4-leaf emission stage (GS14), and on the shoot and root maize biomass at the 5-leaf stage (GS15) in the growth chamber experiment.

Factor	Source of variation	CRI_700_	Chlorophyll (SPAD unit)	NPCI	Biomass (g plant^-1^ d.w.)	
					Shoot	Root
Hybrid (H)	Standard	0.265 a	40.1 a	0.144 b	1.1	1.0
	Early vigor	0.087 b	38.1 b	0.177 a	1.2	1.1
	*p*-value	<0.001	<0.001	0.049	0.126	0.117
Fertilization (F)	Unfertilized	0.225 a	32.3 b	0.118 b	0.4 b	0.6 b
	NP	0.126 b	45.9 a	0.209 a	1.8 a	1.4 a
	*p*-value	0.001	<0.001	<0.001	<0.001	<0.001
Seed treatment (S)	No biostimulant	0.195	39.2	0.146 b	1.0 b	0.9 b
	Biostimulant	0.156	39.0	0.178 a	1.2 a	1.1 a
	*p*-value	0.127	0.632	0.019	<0.001	0.014
H x F	*p*-value	0.008	0.736	0.083	0.592	0.842
H x S	*p*-value	0.257	0.326	0.486	0.186	0.260
F x S	*p*-value	0.440	0.234	0.420	0.002	0.056
H x F x S	*p*-value	0.404	0.988	0.686	0.218	0.231

Means followed by different letters are significantly different. The level of significance (*p*-value) is shown in the Table.

### 3.2 Field experiment

#### 3.2.1 Weather conditions

The meteorological trends observed in each site and in each year are shown in **Table 2**. The two growing seasons showed different weather conditions throughout the maize crop cycle, in particular as far as the rainfall is concerned: the precipitations were more frequent in 2019 than in 2018, above all during the ripening stages (July and August). Carmagnola had a larger amount of rainfall and more air GDDs than Poirino in both years. The soil temperatures, measured from sowing to the 6-leaf stage (GS16), were higher in 2018 than 2019, as a consequence of the later sowing time in both sites.

#### 3.2.2 Early vigor

**Figure 3:**
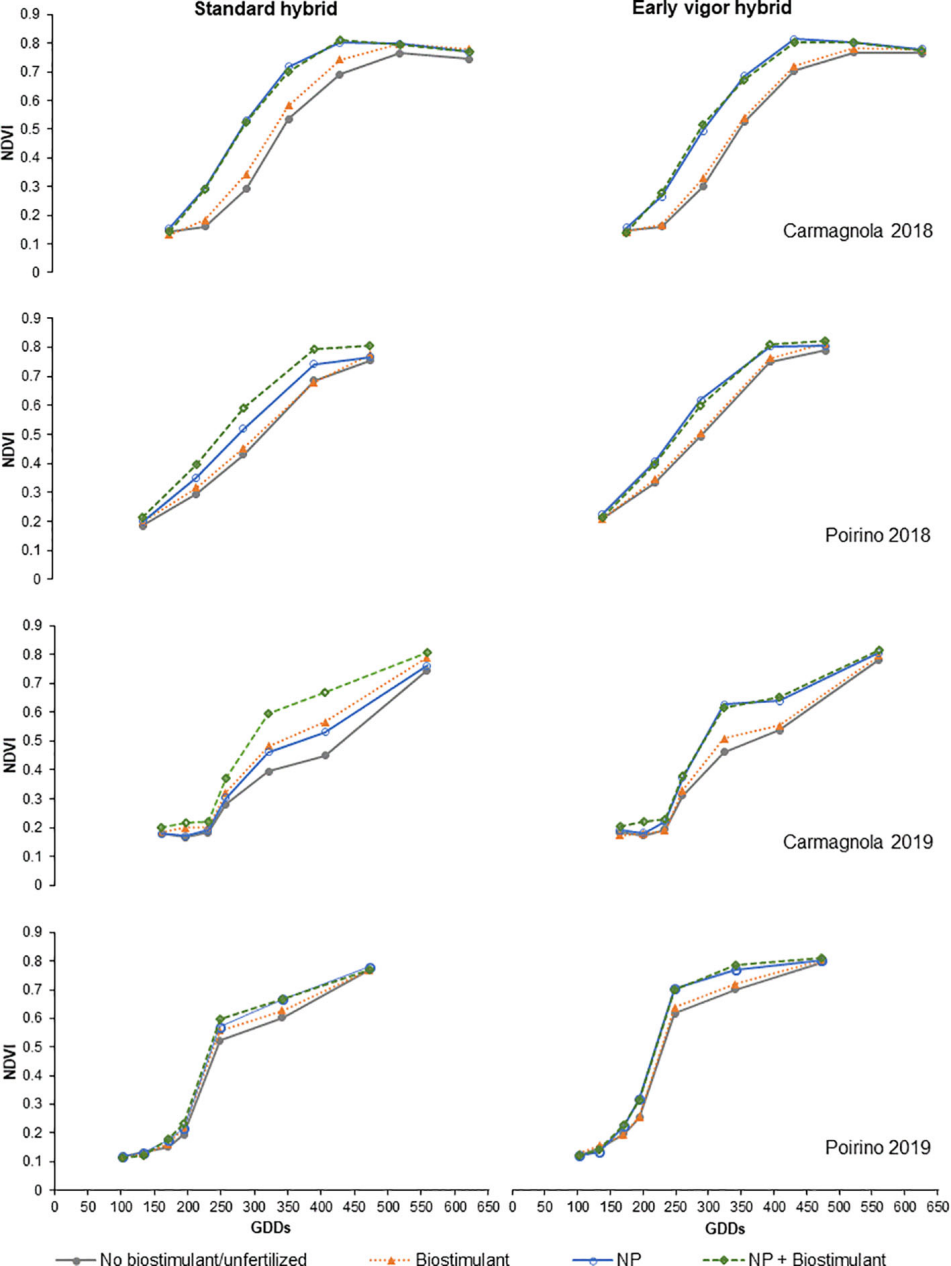
Effects of the hybrid, starter fertilization, and biostimulant seed treatment on the normalized difference vegetation index (NDVI) measured from the 3-leaf stage (GS13) until tassel emission (GS55) in each site (Carmagnola and Poirino) and each year (2018 and 2019). Colors indicate the factorial treatments: no biostimulant/unfertilized (gray), biostimulant (orange), NP starter fertilization (blue) and NP + biostimulant (green). Growing degree days (GDDs): accumulated air growing degree days measured on a 10°C basis.

The NDVI development of the compared treatments, from GS13 to GS55, is reported in **Figure 3**; low values are related to a lower plant biomass and/or greenness status of the maize canopy. Maize growth was faster for the NP starter fertilization than for the unfertilized control in each site and for each year, but a positive effect of the biostimulant seed treatment on plant growth was observed in both sites in 2018 as well as in Carmagnola in 2019. Moreover, these differences were influenced by the maize hybrid: the development of the early vigor maize was faster than that of the standard maize, and the effect of the seed biostimulant and NP fertilization treatments on NDVI development was therefore generally more noticeable for the standard hybrid. A positive synergistic effect on plant development between the starter fertilization at sowing and the biostimulant seed treatment was reported for the control hybrid maize cultivated in Poirino in 2018 and in Carmagnola in 2019. All the NDVI measurements registered during the vegetative growth have been summarized in the AUCDC index (**Table 5**). The NP starter fertilization at sowing resulted in a significant (+13%) increase in AUCDC in both sites and in both years. Both the hybrid and seed biostimulant showed a significant effect on this vegetative index in Poirino in 2018, and at both sites in 2019. The average increase in AUCDC of the early hybrid, compared to the standard one, was 9% in these environments, while the biostimulant application had a 6% greater value than the control (no biostimulant). The interaction between the maize hybrid and seed biostimulant was significant in Carmagnola in 2019: the biostimulant seed application led to a significantly higher development of the plants, albeit only in the standard control hybrid (data not shown). The plant development results, which are summarized by the AUCDC index, were confirmed by considering the height of the plants measured at GS14 and GS33. The early vigor hybrid significantly increased (+30%) the plant height at the leaf emission stage in all the environments, compared to the standard maize, with the exception of the experiment carried out in 2018 in Carmagnola. No significant differences between hybrids were observed at the stem elongation stage. The NP starter fertilization had a significant effect on plant vigor in all the experiments and at both growth stages. On average, the plant height increased by 43% and 90%, compared to the unfertilized control, at GS14 and GS33, respectively. The biostimulant seed treatment only increased the plant height significantly (+22%) at GS33, although no effect was reported in the experiment carried out in 2019 in Poirino. The interactions between factors were never significant for the plant height parameter at GS14. Conversely, a significant starter fertilization × seed biostimulant interaction was reported in Carmagnola in 2018 at GS33, where the biostimulant seed treatment led to a significantly higher development of the plants, albeit only in the unfertilized control (data not shown). Crop development differences were also detected between the compared factors in the later growth stages, at flowering and at harvest (**Table 6**). The hybrid and the NP starter fertilization significantly affected the flowering date in all the experiments. The use of the NP fertilization at sowing and the early vigor hybrid, instead of the standard one, led to an earlier flowering date, that is, of 2.1 and 2.8 days, respectively. Furthermore, the NP fertilization showed a more consistent effect on the grain moisture content at harvest: the NP starter fertilizer significantly reduced the moisture content, compared to the unfertilized control, on average by 1.1 percentage points, in all the experiments. The hybrid with an early vigor was instead harvested with a significantly lower moisture content than the standard one in the experiment carried out in Poirino in both years. A significant anticipation of the flowering date (-0.8 days) was observed for the biostimulant seed treatment, albeit only in the experiment carried out in Poirino in 2018, while the seed treatment did not affect the grain moisture at harvest in any experiment. A significant interaction between hybrid and seed treatment was reported for the flowering date in 2018 in Carmagnola: the biostimulant only led to an anticipated flowering in the early vigor hybrid (data not shown). The interaction between the fertilization and the seed treatment was significant for the grain moisture at harvest in both sites in 2018. The application of a seed biostimulant decreased the grain moisture by 1 percentage point in the unfertilized control in Carmagnola and by 2 percentage points in Poirino, where the NP starter fertilizer was applied, respectively (data not shown).

**Table 5:** Effects of the hybrid, starter fertilization, and biostimulant seed treatment on the early vigor of maize, expressed as the area under the canopy development curve (AUCDC) and the plant height at the leaf emission (GS14) and stem elongation (GS33) stages for the Carmagnola and Poirino field experiments in the 2018 and 2019 growing seasons.

Factor	Source of variation	AUCDC (Σ NDVI-day)				Plant height GS14 (cm)				Plant height GS33 (cm)			
		2018		2019		2018		2019		2018		2019	
		Carmagnola	Poirino	Carmagnola	Poirino	Carmagnola	Poirino	Carmagnola	Poirino	Carmagnola	Poirino	Carmagnola	Poirino
Hybrid (H)	Standard	23.6	20.3 b	19.9 b	19.9 b	15.9	17.5 b	6.9 b	11.3 b	70.9	55.6	23.4	49.1
	Early vigor	23.0	21.6 a	21.2 a	23.0 a	16.0	20.3 a	8.8 a	16.5 a	62.7	63.5	25.4	53.5
	*p*-value	0.212	<0.001	0.001	<0.001	0.417	<0.001	<0.001	<0.001	0.223	0.106	0.096	0.126
Fertilization (F)	Unfertilized	21.0 b	19.9 b	19.1 b	20.6 b	12.3 b	16.0 b	6.4 b	12.2 b	45.6 b	42.6 b	14.0 b	41.1 b
	NP	25.4 a	21.8 a	21.7 a	22.1 a	19.9 a	21.3 a	9.3 a	15.9 a	85.5 a	76.0 a	34.0 a	61.5 a
	*p*-value	<0.001	<0.001	<0.001	<0.001	<0.001	<0.001	<0.001	<0.001	<0.001	<0.001	<0.001	<0.001
Seed treatment (S)	No biostimulant	22.9	20.5 b	19.4 b	21.1 b	15.7	18.5	7.4	13.7	62.0 b	54.1 b	21.1 b	50.3
	Biostimulant	23.7	21.3 a	21.6 a	21.6 a	16.3	19.0	8.2	14.4	72.2 a	63.7 a	28.1 a	52.4
	*p*-value	0.084	0.034	<0.001	0.034	0.494	0.195	0.076	0.215	0.018	0.032	0.015	0.618
H x F	*p*-value	0.688	0.473	0.241	0.065	0.489	0.102	0.091	0.215	0.639	0.152	0.482	0.006
H x S	*p*-value	0.933	0.128	0.001	0.754	0.763	0.775	0.186	0.863	0.395	0.670	0.292	0.805
F x S	*p*-value	0.053	0.632	0.549	0.277	0.080	0.660	0.972	0.326	0.033	0.563	0.164	0.923
H x F X S	*p*-value	0.843	0.142	0.369	0.777	0.302	0.444	0.983	0.977	0.349	0.993	0.181	0.657

Means followed by different letters are significantly different. The level of significance (*p*-value) is shown in the Table.

**Table 6:** Effects of the hybrid, starter fertilization, and biostimulant seed treatment on the early vigor of maize, expressed as the date of flowering (Days after sowing, DAS) and grain moisture at harvest for the Carmagnola and Poirino field experiments in the 2018 and 2019 growing seasons.

Factor	Source of variation	Date of flowering (DAS)				Grain moisture (%)			
		2018		2019		2018		2019	
		Carmagnola	Poirino	Carmagnola	Poirino	Carmagnola	Poirino	Carmagnola	Poirino
Hybrid (H)	Standard	75.9 a	75.1 a	105.7 a	108.2 a	22.7	24.3 a	25.2	28.7 a
	Early vigor	74.3 b	72.7 b	101.8 b	104.9 b	23.2	23.1 b	25.2	26.3 b
	*p*-value	<0.001	<0.001	<0.001	<0.001	0.334	0.008	0.934	<0.001
Fertilization (F)	Unfertilized	76.4 a	74.8 a	104.7 a	107.6 a	23.5 a	24.3 a	25.9 a	28.0 a
	NP	73.8 b	72.9 b	102.9 b	105.4 b	22.4 b	23.2 b	24.6 b	26.9 b
	*p*-value	<0.001	<0.001	<0.001	<0.001	0.029	0.017	0.023	0.004
Seed treatment (S)	No biostimulant	75.3	74.3 a	103.9	106.4	23.1	24.1	25.4	27.4
	Biostimulant	74.9	73.4 b	103.7	106.6	22.8	23.4	25.0	27.5
	*p*-value	0.237	0.003	0.586	0.948	0.479	0.103	0.500	0.883
H x F	*p*-value	0.039	0.028	0.586	0.473	0.485	0.783	0.898	0.394
H x S	*p*-value	0.039	1.000	0.459	0.948	0.654	0.454	0.744	0.336
F x S	*p*-value	0.237	0.643	0.303	0.743	0.011	0.045	0.631	0.458
H x F X S	*p*-value	0.810	1.000	0.520	0.647	0.840	0.472	0.485	0.542

Means followed by different letters are significantly different. The level of significance (*p*-value) is shown in the Table.

#### 3.2.3 Grain yield and yield parameters

The NP fertilization at planting significantly increased the grain yield in all the experiments, although a significant effect of hybrid and the seed treatment was only reported for Poirino in 2018 and Carmagnola in 2019 (**Table 7**). On average, the starter NP fertilization increased the grain yield, compared to the unfertilized control, by 6%, while the average yield enhancement values obtained for adopting an early vigor hybrid, instead of the standard one, and for the application of seed biostimulant, instead of no biostimulant control, were 8.5% and 5.1%, respectively. The grain yield increase was mainly related to an enhancement of KSM for all the factors, while the hybrid and NP fertilization significantly affected the TKW, albeit only in Poirino in 2018. In this experiment, the higher KSM that was observed for the early vigor hybrid and NP starter fertilization led to a significantly, but less proportional, lower TKW, although a significant yield increase was maintained. The interaction between the involved agronomic factors for grain yield and yield components was only significant under a few of the considered conditions. The NP starter fertilization led to a higher grain yield in the early vigor hybrid in Poirino in 2018 and in Carmagnola in 2019.

**Table 7:** Effects of the hybrid, starter fertilization, and biostimulant seed treatment on the maize grain yield and the yield component, kernels per square meter (KSM), and thousand kernel weight (TKW) for the Carmagnola and Poirino field experiments in the 2018 and 2019 growing seasons.

Factor	Source of variation	Grain yield (t ha^-1^)				KSM (n° m^−2^)				TKW (g)			
		2018		2019		2018		2019		2018		2019	
		Carmagnola	Poirino	Carmagnola	Poirino	Carmagnola	Poirino	Carmagnola	Poirino	Carmagnola	Poirino	Carmagnola	Poirino
Hybrid (H)	Standard	17.1	16.1 b	14.9 b	15.0	4850	4902 b	4190 b	4301	401	418 a	369	388
	Early vigor	17.0	17.2 a	16.4 a	15.6	4764	5205 a	4796 a	4321	393	400 b	367	384
	*p*-value	0.941	0.001	<0.001	0.101	0.361	0.036	<0.001	0.798	0.111	<0.001	0.674	0.514
Fertilization (F)	Unfertilized	16.6 b	16.2 b	15.2 b	14.9 b	4425 b	4858 b	4163 b	4164 b	396	412 a	367	386
	NP	17.7 a	17.1 a	16.1 a	15.7 a	5287 a	5248 a	4823 a	4471 a	398	407 b	370	385
	*p*-value	<0.006	0.004	0.021	0.032	<0.001	0.011	<0.001	0.028	0.637	0.019	0.530	0.865
Seed treatment (S)	No biostimulant	16.8	16.3 b	15.2 b	15.1	4847	4902 b	4392 b	4281	401	410	372	386
	Biostimulant	17.4	17.0 a	16.1 a	15.5	4766	5205 a	4627 a	4341	393	409	365	386
	*p-*value	0.312	0.004	0.005	0.344	0.221	0.036	0.031	0.543	0.142	0.628	0.220	0.952
H x F	*p*-value	0.080	0.003	0.018	0.211	0.852	0.409	0.052	0.078	0.328	0.123	0.573	0.419
H x S	*p*-value	0.752	0.124	0.124	0.829	0.023	0.572	0.224	0.402	0.466	0.021	0.155	0.358
F x S	*p*-value	0.067	0.239	0.631	0.972	0.025	0.476	0.475	0.507	0.988	0.004	0.835	0.784
H x F X S	*p*-value	0.357	0.906	0.026	0.664	0.901	0.748	0.139	0.942	0.970	0.195	0.058	0.771

Means followed by different letters are significantly different. The level of significance (*p*-value) is shown in the Table.

## 4 Discussion

This study provides useful data that can be used to compare the agronomic and grain yield benefits of different crop practices that are able to enhance the early plant vigor of maize, which is considered, in temperate growing areas, a key stage to maximize the production and thus increase the overall sustainability of maize cropping systems. The effects of the maize genotype, the NP starter fertilizer, and the biostimulant seed treatment on the early stages of maize development, and the consequent effect on the length of the crop cycle, as well as on the grain yield and quality, were studied in depth in both a growth chamber experiment and open field experiments, considering the factorial combination of the compared agronomic factors in different locations and different growing seasons.

First, this experiment has allowed a direct comparison to be made of the efficacy of the tested crop practices in enhancing plant vigor. The starter fertilization, which involved the distribution of N and P in the furrows at sowing, led to the greatest and steadiest effect, in terms of plant development and final grain yield, in both the growth chamber experiment and the open field experiments. The effect on plant vigor of the starter fertilization, or the use of a vegetative index, such as NDVI, from the 3-leaf growth stage, was evident across the growth stage assessments in both the field experiments. In agreement with Ma et al. (2013), the positioning of N and P close to plants led to the early root growth, and hence, the nutrient uptake being increased, which in turn influenced the plant morphology and physiology by creating a larger leaf area. This resulted in a greater radiation interception throughout the entire maize growing cycle. Such an increased leaf area was closely correlated with a high rate of photosynthesis and a high chlorophyll content (Rostami et al., 2008), as suggested by the SPAD and NPCI values. A starter fertilization is primarily aimed at satisfying the crop P uptake. Moreover, this experiment confirms that the effect of P distribution at sowing also enhances the early vigor of maize in soils (see, for example, the Poirino site), where this macronutrient is present in large quantities, thus confirming that the cold meteorological conditions of an early sowing could limit its absorbance, even in agronomic conditions where this element is abundant (Roth et al., 2006). Furthermore, N and P subsurface banded together showed a positive and synergistic interaction on nutrient uptake and plant growth, in comparison with N (Blandino et al., 2022) or P (Jing et al., 2010) on their own. A better early growth and early establishment allowed the flowering of maize to be anticipated, thereby reducing the interval between sowing and silking, making the interception of the solar radiation within the growing season more efficient and increasing the final grain yield (Tsimba et al., 2013). The starter fertilization always significantly impacted the duration of the crop cycle in both experimental fields, since it shortened the time from sowing to flowering, and it decreased the grain moisture of the maize at harvest (Kaiser et al., 2016). Thus, the possible advantages for the maize cropping system are not only linked to a lower drying cost, but also to the possibility of harvesting in advance, thereby reducing the mycotoxin contamination risk (Blandino et al., 2009), or of using a later hybrid with the same growth stage duration, in order to enhance the grain yield potential (Tsimba et al., 2013). Furthermore, an earlier sowing, combined with the use of the agronomic practices able to reduce the sowing-flowering period, can reduce the adverse effects of climate change in terms of drought and high temperature stresses, which could heavily affects maize at flowering (Kafaie Ghaeini et al., 2023).

Overall, a starter fertilization is expected to stimulate a faster root system establishment, which could lead to a more effective uptake of all the nutrients by the crop. In addition to the direct effect on plant growth, the first part of the present study (Ujvári et al., 2023) highlighted that the application of an NP fertilizer could modify the rhizosphere bacterial community to a great extent, and thus positively increase several richness and diversity indices. Moreover, the rise in the availability of mineral nutrients and the higher production of root exudates, as a consequence of a higher plant development (Zhu et al., 2016), showed an increase in the occurrence of specific genera (e.g., *Stenotrophomonas* and *Lysobacter*) which are P solubilizers (Ghosh et al., 2020; Dai et al., 2023), thus leading to a further improvement in the nutrient use efficiency.

The early vigor hybrid showed a more rapid crop establishment in the earliest vegetative growth stages, and this resulted in a significant anticipation of the crop cycle and an increase in the grain yield, compared to the standard one, in half of the considered production situations. Maize breeding has produced an increase in tolerance to low temperatures in modern hybrids, in order to reduce the physiological impacts of cold temperatures, which could limit the uptake of nutrients by the root system and plant photosynthesis (Haldimann, 1999; Gillani et al., 2021). The data collected in the growth chamber experiment, carried out at a low temperature, which is typical of an early sowing time, highlighted that the standard hybrid showed more nutritional stress symptoms, with more yellow/orange leaves, than the early vigor one. These symptoms are related to a higher occurrence of carotenoid (Li et al., 2008) and anthocyanin (Pietrini et al., 2002) compounds in the leaves, which play a precursor signaling role of plant stress defense. The early vigor hybrid showed significantly higher LA values than the standard one, already from the 2-leaf stage. An early larger leaf area allows plants to capture sunlight more effectively during early canopy development (Wijewardana et al., 2016), thereby increasing the photosynthesis rate, which could help support a further, more rapid plant growth. In addition to the effect of epigeal development, the root system might also be affected by a more rapid growth, with a more extended root volume and a higher root exudation of organic acids being able to increase the nutrient availability and uptake (Hund et al., 2008). Furthermore, the root traits, in particular the production of root exudates, could influence the microbial rhizosphere community, as reported in part I of the present study (Ujvári et al., 2023), in which a clear difference in the composition of the microbiota was reported between the two maize hybrids. Although the richness of the bacterial species was slightly higher in the standard hybrid at plant emergence, both the richness and the Hill^2^ biodiversity indices were much higher in the high vigor hybrid at the 5-leaf stage.

The biostimulant seed treatment showed a significant effect on several plant growth indices in both the growth chamber and the field experiments. Furthermore, this treatment was less effective in reducing the interval between sowing to flowering and in lowering the moisture content of the grain at harvest, although a significant increase in grain yield was detected in 2 of the 4 compared production situations. As already reported in the literature (Mickan et al., 2021), the biostimulant effect of adding microbial inoculants may vary, according to the environmental and agronomic conditions. Several studies have underlined that biostimulants can play a role under conditions of great crop stress (Schütz et al., 2018; Li et al., 2022). In our field trials, the biostimulant seed treatment was more effective in enhancing plant growth and grain yield in the experiments carried out with the coldest soil temperatures from sowing to the 6-leaf stage (e.g. Poirino 2018 and Carmagnola 2019). Although beneficial microorganisms have been used largely as inoculants for crops, their agronomical benefits have generally been reported in marginal environments, with a low yield potential (Li et al., 2022), while their contribution to intensive cropping systems has not yet been clarified. Instead, in this study, the agronomic advantages of a biostimulant, based on a PGPB strain, has been reported in production situations with high soil fertility and a high yield potential, but focusing on a specific critical factor that could limit plant productivity.

A *B. amyloliquefaciens* seed application response in wheat (Donn et al., 2015; Wolińska et al., 2020) has also been reported to be influenced to a great extent by its interaction with other crop practices, such as the genotype and fertilization. In the present study, the best advantage of the seed biostimulant, in terms of NDVI value, was reported for the standard hybrid, which is more prone to environmental and nutritional stress. Although the DGGE analysis did not detect an increase in the occurrence of this species in the maize rhizosphere (see Part I), a contribution of this PGPB, especially in the first growth stage, should not be excluded. *B. amyloliquefaciens* is one of the most efficient bacteria, and it is able to solubilize organic and inorganic P (Nkebiwe et al., 2017; Mpanga et al., 2020), and to release auxin and ACC deaminase (Borriss, 2015). Moreover, in part I of the work, the biostimulant seed treatment affected the composition of the rhizosphere bacterial community of the two hybrids in different ways, and showed a greater effect on the standard one. The application of the biostimulant led to a more abundant occurrence of the *Paenibacillus* and *Stenotrophomonas* species, which are well known for their wide range of plant growth promoting properties (N2 fixation, P solubilization, as well as plant hormone and siderophore production). Hui et al. (2018) reported that the application of *B. amyloliquefaciens* to soil led to an initial increase in the concentration of nitrate, which affected the soil microbial community composition. *B. amyloliquefaciens* seed inoculation has in particular been shown to promote the abundance of the rhizosphere microorganisms involved in the soil nutrient cycles (Luo et al., 2022). *B. amyloliquefaciens* has also been shown to be able to directly influence the plant roots of *Arabidopsis thaliana*, changing their structure by increasing lateral outgrowth and elongation, and root-hair formation, thus promoting plant growth (Asari et al., 2017). In addition to the close relationship between the maize hybrids and the biostimulant seed treatment, a synergistic effect between the biostimulant and the NP starter fertilizer was observed in both experiments. The biostimulant, in combination with the NP fertilizer, determined a faster growth rate for each maize hybrid, especially the standard one. Since it was applied to the seed, the biostimulant may have produced an early growth-promoting effect on the seedling during germination, thus allowing the roots to reach the fertilizer localized in the seed furrow more quickly and to anticipate its benefits. Moreover, the effect on the microbiota composition, that is, an increase in PGPB that was able to solubilize nutrients, may have improved the maize plants’ use of the starter fertilizer, which resulted in greater availability of such macronutrients (Xue et al., 2021). Thus, the role of biostimulants in cropping systems should not only be considered as an eco-friendly alternative to applying fertilizers, but also as a solution to improve the effectiveness of fertilizing practices, especially when using a high-efficiency strategy, such as its application to seed furrows. However, this synergistic effect requires further studies to evaluate how to maintain the same effectiveness on the early vigor of maize but limiting the quantity of applied N and P, especially to soils with high P availability, where the need of an additional application is questionable (Schröder et al., 2015).

**Figure 4:**
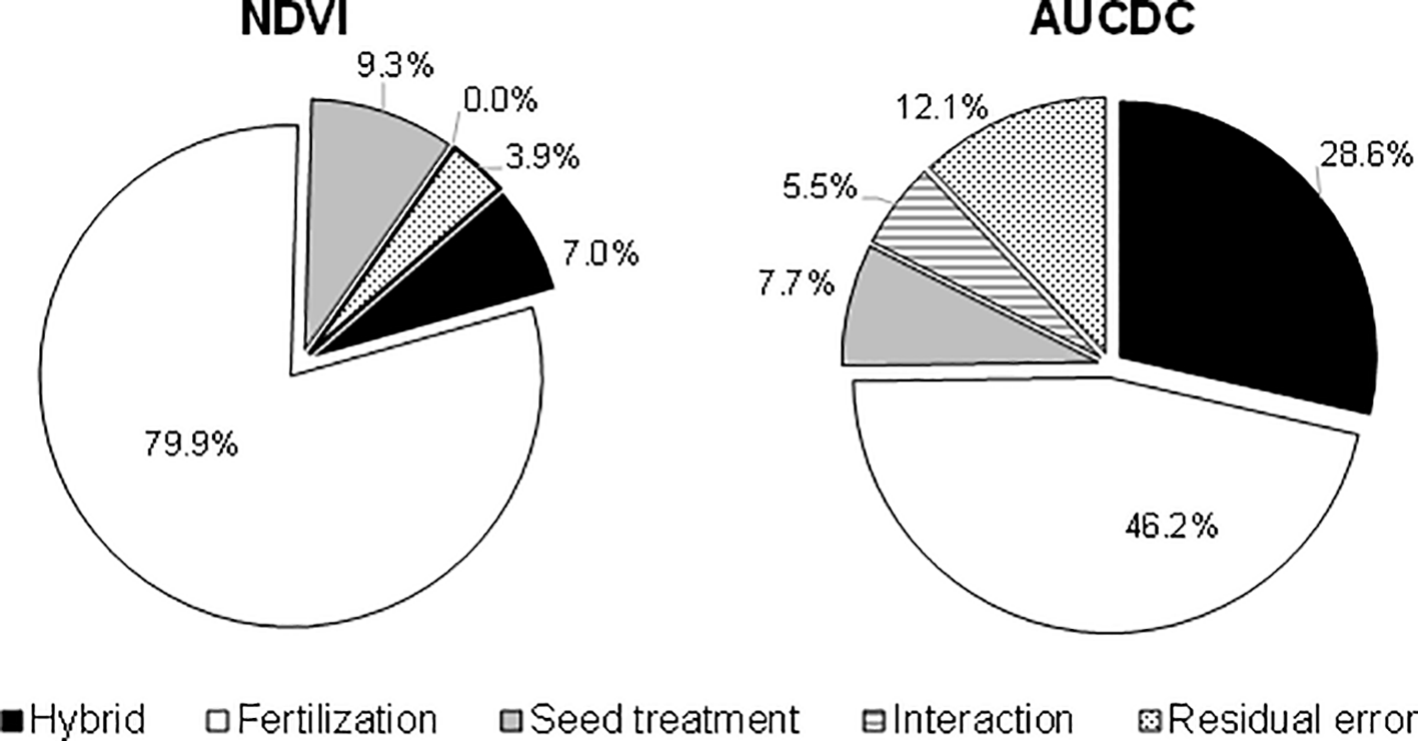
Variance components of the NDVI index at the 5-leaf stage (growth chamber experiment) and of the AUCDC index (field experiments). The variance components were calculated as the ratio of the variance of each agronomic factor to their interaction on the total variance of ANOVA. The data on AUCDC refer to ANOVA, as applied to all 4 field experiments.

**Figure 5:**
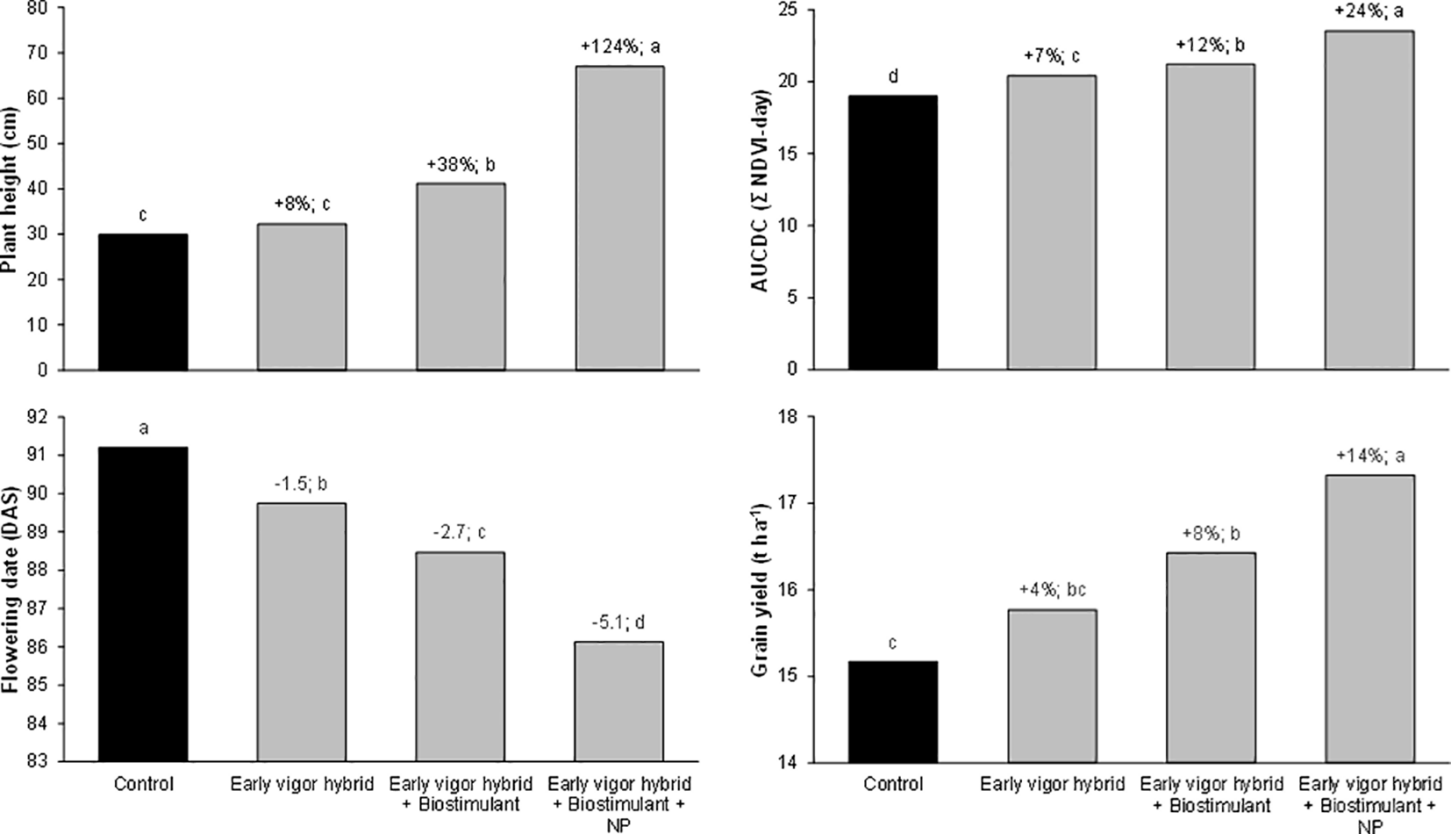
Effect of the hybrid, starter fertilization, and biostimulant seed treatment on the early vigor of maize, expressed as plant height at the stem elongation stage (GS33), the area under the canopy development curve (AUCDC), the flowering date, and the grain yield. Bars with different letters are significantly different (*p*-value< 0.05), according to the REGW-F test. The reported values are based on 16 observations (2 locations X 2 years X 4 replicates). Control: standard hybrid (LG30600) with no biostimulant or NP starter fertilizer. Early vigor hybrid: LG31630 with no biostimulant or NP starter fertilizer. Early vigor hybrid + biostimulant: LG31630 with a biostimulant seed treatment (mixture of a bacterium, *Bacillus amyloliquefaciens* IT-45 strain (Rise P®), and a leguminous plant extract *Cyamopsis psoraloides* (AgRho^®^ GSB30). Early vigor hybrid + biostimulant + NP: LG31630 with a biostimulant seed treatment and the distribution of 27 kg N ha^-1^ and 69 kg P2O5 ha^-1^ as diammonium phosphate at sowing.

In addition to the single effect of each treatment, the full factorial combination of the considered agronomic factors has allowed a direct comparison to be made of their importance and of the benefits of their combination on enhancing the initial plant growth across the whole cropping system. The influence of the three agronomic factors (hybrid, starter fertilization, and seed treatment) and their interaction on the NDVI value detected at the 5-leaf stage in the growth chamber and on AUCDC (sum of the NDVI value detected for the 3-leaf stage at tassel emission) in the field experiments were evaluated by means of three-way ANOVA (**Figure 4**). In both experiments, the starter fertilization accounted for the highest percentage of variation, in particular in the growth chamber experiment where maize growth was limited to the 5-leaf stage. The use of hybrids with different early vigor explained the lower amount of variation in the growth chamber experiment, while it accounted for 29% of the total variation in the field trials. The biostimulant seed treatment accounted for 8-9% of the total variation in both experiments. Overall, the interaction between the involved agronomic factors explained less than 6% of the total variation in the early growth of the plants. Furthermore, the collected data highlighted some significant interactions among these practices, and it was considered interesting to address the overall benefits of their different combined application within the maize cropping system. With this aim, the advantages, in terms of plant vigor, the shortening of the sowing – flowering interval, and grain yield, are summarized in **Figure 5**, taking into account some of the possible cropping systems and comparing them with a control situation (a standard maize hybrid with no seed biostimulant or starter NP fertilizer): I) an early vigor hybrid; II) an early vigor hybrid plus the biostimulant seed treatment; and III) the combination of the above mentioned two strategies together with NP localized fertilization at sowing. Compared to the use of an early vigor hybrid, its combination with the biostimulant seed treatment led to a significant further enhancement of plant growth and a reduction of the sowing-flowering period. The combination of all 3 factors resulted in the maximum benefits, compared to the control cropping system, with a 124% increase in the plant growth, a 5-day reduction of the sowing-flowering period and a 14% gain in grain yield.

In the context of the development of future cereal cropping system, considering the introduction of environmentally-friendly innovations in a sustainable intensification approach, this study has highlighted the importance of innovating the crop practices characterized by a high input use efficiency. As far as the maize cropping system is concerned, the application of agronomic techniques that are able to promote plant vigor in the early vegetative stages is a key factor that leads to significant and sustainable yield advantages. Here, the starter fertilization, with the application of N and P close to seed furrows, had the greatest effect, thus suggesting that simply reducing fertilizer inputs may represent a significant drawback for high N- and P-requiring crops, such as cereals, and that it is necessary to re-design the fertilization strategies by above all enhancing the input use efficiency, focusing on the most critical growth stages for nutrient uptake. As far as alternative practices are concerned, the use of innovative genotypes, with early vigor, or biostimulant seed applications, are crop techniques that can attenuate the abiotic stress factor of an early sowing, thus leading to a prompter crop development. These innovations can therefore represent a solution that can be used to reduce or, in certain cases, replace a starter fertilization. Furthermore, the study has highlighted a positive additive effect of these practices, that is, of further increasing the initial plant vigor and the associated agronomic advantages. The choice of the most suitable crop practices should consider the diversity of each cropping system, according to the pedo-climatic conditions, the agronomic background, the yield potential, and the requirements of the supply chain. Moreover, in a more holistic view, the present study (see part I, Ujvári et al., 2023) has highlighted that the crop practices evaluated in our work positively influenced the rhizosphere microbiota composition, thereby playing a clear role in the management of microbial soil fertility and leading to a possible further contribution, that is, favoring the early development of maize. These findings indicate the need to study the complexity of the interactions between crop practices and plant microbiota in more depth, in order to define the most suitable cropping systems to maximize the profitability and sustainability of maize cultivation in different production situations.

## Data availability statement

The raw data supporting the conclusions of this article will be made available by the authors, without undue reservation.

## Author contributions

This work was conceptualized and supervised by LC, MA, and MB. Methodology and investigation was done by LC, AS, GU, and MB. The original draft was written by LC and MB. All authors contributed to the article and approved the submitted version.
