# Supplementary material for: Agronomic strategies to enhance the early vigor and yield of maize part II: the role of seed applied biostimulant, hybrid, and starter fertilization on crop performance
Source: Front Plant Sci. 2023 Nov 3;14:1240313. doi: 10.3389/fpls.2023.1240313 (PMC10656683; doi:10.3389/fpls.2023.1240313)
Supplement: Supplementary file 1 [file Table_1.docx]

Supplementary Material

Agronomic strategies to enhance the early vigor and yield of maize. Part II: the role of seed applied biostimulant, hybrid, and starter fertilization on crop performance

Luca Capo^1^, Alessandro Sopegno^1^, Amedeo Reyneri^1^, Gergely Ujvári^2^, Monica Agnolucci^2^*, Massimo Blandino^1^*

^1^Department of Agriculture, Forest and Food Sciences, University of Turin, Grugliasco, Italy

^2^Department of Agriculture, Food and Environment, University of Pisa, Pisa, Italy

*** Correspondence:**

Massimo Blandino: [massimo.blandino@unito.it](mailto:massimo.blandino@unito.it)

Monica Agnolucci: [monica.agnolucci@unipi.it](mailto:monica.agnolucci@unipi.it)

# Supplementary Table

**Table S1.** The main agronomic information pertaining to the maize growing cycle in the growth chamber experiment.

| Medium temperature of the air | Day | 16.7 | C° |
| --- | --- | --- | --- |
|  | Night | 14.1 | C° |
| Air GDDs ^1^ | Sowing - 6 leaves | 273 | C°-day |
| Medium temperature of the soil | Day | 15.6 | C° |
|  | Night | 14.2 | C° |
| Soil GDDs | Sowing - 6 leaves | 237 | C°-day |
| Air Humidity | | 50 | % |
| Day/night | | 12 | h |
| Light intensity | | 700-1000 | PAR ^2^ |
| Water irrigation | | 10 mm every 7 day | |

^1^ GDDs: accumulated growing degree days on a 10°C basis.

^2^ PAR: photosynthetically active radiation: μmol m^-2^ s^-1^
